# Do Patients With Higher Preoperative Functional Outcome Scores Preferentially Seek Direct Anterior Approach Total Hip Arthroplasty?

**DOI:** 10.1016/j.artd.2021.05.018

**Published:** 2021-06-22

**Authors:** Wayne E. Moschetti, Samuel Kunkel, Benjamin J. Keeney, David Jevsevar

**Affiliations:** a Department of Orthopaedics, Geisel School of Medicine, Dartmouth-Hitchcock Medical Center, Lebanon, NH, USA; b Berkley Medical Management Solutions, Overland Park, KS, USA

**Keywords:** Total hip arthroplasty, Direct anterior approach, Posterior approach, PROMIS-10, Health related quality of life

## Abstract

**Background:**

There is focus on the direct anterior approach (DAA) for total hip arthroplasty because of perceived postoperative functional improvement. We compared baseline, short-term, and long-term outcomes between the DAA and the posterior approach focusing on baseline function.

**Material and methods:**

Multivariate linear and logistic regression models were used to analyze prospective data on 1457 total hip arthroplasties comparing baseline characteristics, operative time, 90-day reoperation, length of stay (LOS), extended LOS (>3 days), and facility discharge. The Patient-Reported Outcome Measurement Information System-Global Health (PROMIS-10) was used to determine physical component score (PCS) and mental component score (MCS), with clinically significant improvement defined as >5 points. Adjusters included age, sex, race/ethnicity, year, Charlson Comorbidity Index, body mass index, alcohol, and tobacco use.

**Results:**

DAA patients had higher preoperative MCS (DAA 50.4 vs posterior approach 47.4, *P* < .001), PCS (40.7 vs 38.5, *P* < .001), and postoperative PCS scores (48.9 vs 46.7, *P* < .001). There was no difference in mean PCS improvement (8.1 vs 8.2; *P* = .798) or clinically significant PCS change (*P* = .963). DAA was associated with shorter LOS by 0.49 days (95% confidence interval [CI] = 0.32-0.65, *P* < .001), lower odds of extended LOS (odds ratio = 0.33, 95% CI = 0.21-0.50, *P* < .001), and lower odds of facility discharge (odds ratio = 0.54, 95% CI = 0.37-0.79, *P* < .001). No difference in operative time (86 vs 87 minutes; *P* = .812) or 90-day reoperations (1 vs 1%; *P* = .347) was observed.

**Conclusion:**

DAA patients presented with higher preoperative PCS and MCS scores, yet both groups experienced significant improvement. DAA was associated with decreased LOS and lower odds of extended LOS and facility discharge. There was no difference in operative time or reoperation.

## Introduction

Total hip arthroplasty (THA) is a common procedure for the treatment of end-stage degenerative arthritis of the hip that has failed nonoperative management [1]. Despite the success of THA, there remains a continued focus on operative techniques to further improve patient outcomes and minimize complications. Surgical approach is an area which has gained attention for perceived benefits after THA. There are several surgical approaches by which THA can be performed, and there are reported merits and risks to each [[2], [3], [4], [5], [6]].

Over the past decade, the direct anterior approach (DAA) has become increasingly popular in the United States for primary THA [7,8]. This approach uses a true intermuscular plane and may minimize abductor dysfunction postoperatively by avoiding iatrogenic injury to the gluteus maximus, tensor fascia latae, and gluteus medius. Proponents of this approach believe that by sparing the posterior and lateral hip musculature during surgical dissection, patients may recover faster with fewer functional limitations, less dependence on assistive devices, and lower dislocation risk [[9], [10], [11], [12], [13], [14], [15], [16], [17], [18], [19], [20], [21]]. Moreover, patients are typically positioned supine for this approach, which affords the opportunity for easy access to intraoperative fluoroscopy, potentially facilitating improved acetabular component positioning [22].

Despite the successful use of DAA for THA, concerns remain for differences in complication rates and functional outcomes compared with the posterior approach (PA) [[23], [24], [25]]. While prior literature has suggested that surgeons who are facile with DAA technique may perform the operation safely and that there may be some short-term benefits to this approach, there remains a paucity of comparative data on global health-related quality of life (HRQoL) improvement after this surgery. Most studies have focused on disease-specific outcomes or functional metrics such as the use of walking aids [16,20,26,27].

The goal of this study was to evaluate baseline, perioperative, and short- and long-term differences in global HRQoL patient-reported outcomes for patients undergoing THA through either the DAA or PA. In addition, we sought to explore the differences in hospital length of stay (LOS) and discharge disposition in these patient cohorts.

## Material and methods

The study protocol was reviewed by our institutional review board, and a waiver of informed consent was obtained. All surgeries were performed by 6 experienced surgeons who routinely perform THA exclusively through either the DAA or PA, at a tertiary academic medical center. Any surgeon who performed both approaches was removed from the analysis to avoid surgeon-patient selection bias. Multivariate linear and logistic regression techniques were used to analyze prospectively collected data, adjusting for preoperative clinical and demographic variables. Regression modeling was used to determine the association of the DAA and PA with LOS, extended LOS (>3 days), facility discharge disposition, physical function improvement, clinically significant physical function improvement (>5 points), operative time in minutes, and 90-day any-type reoperation [28,29]. Clinical and demographic variables assessed included age, sex, race/ethnicity, year, Charlson Comorbidity Index, prior contralateral primary THA, patient-reported Patient-Reported Outcome Measurement Information System-Global Health (PROMIS) physical component score (PCS) and mental component score (MCS), timing of postoperative PCS collection, body mass index (BMI), and alcohol and tobacco use.

PCS and MCS were determined by standardized PROMIS-10 and Veterans RAND-12 (VR-12) as markers of HRQoL. If PROMIS-10 was unavailable, we converted VR-12 scores to PROMIS-10 scores using the method outlined by Schalet et al [30]. When both VR-12 and PROMIS-10 scores were available for the same patient, at the same time point, the PROMIS-10 score was used. If multiple postoperative time periods were captured, then the “priority” was as follows: 300-420 days (1 year), 421+ days, 46-299 days, and 0-45 days.

We used t-tests for continuous comparisons and chi-square for categorical comparisons. A *P*-value below 0.05 was considered to be significant. Analysis was performed using Stata 15 MP (StataCorp, College Station, Texas, 2017).

## Results

From April 2011 through July 2016, 6 surgeons performed 1457 THAs among 1353 individuals. The data included 1052 DAA THAs (reference group) and 405 PA THAs. Baseline clinical and demographic characteristics of our sample are provided in Table 1. There was no significant difference in the mean age of patients between the groups (DAA 63.3 [standard deviation = 11.5] vs PA 64.6 [standard deviation = 12.6]; *P* = .062) or sex (53% female patients undergoing DAA vs 52% female patients undergoing PA; *P* = .660). The vast majority of patients were non-Hispanic white, reflecting our local population, and there was no difference in race/ethnicity between groups (98 vs 98%; *P* = .356). The patients in the PA group had higher Charlson Comorbidity Scores (*P* < .001). There was also a small but statistically significant difference in BMI between the PA group (30.1) and the DAA group (29.1) (*P* = .01). There was no difference between groups in mean operative time (86 minutes vs 87 minutes; *P* = .812) or 90-day reoperation (0.7% PA vs 1.3% DAA; *P* = .347).

**Table 1:** Counts and bivariate analyses of relevant variables among posterior and anterior total hip arthroplasty patients.

Variable	Posterior approach, N (405, 28%)	Anterior approach, N (1052, 72%)	Posterior %	Anterior %	*P* value
Age mean years (SD, range)	64.6 (12.6, 18.7 to 91.3)	63.3 (11.5, 18.9 to 95.8)			.062
Age Group (ref = <55)	77	223	19	21	.033
55-59	51	162	13	15	
60-64	81	201	20	19	
65-69	57	184	14	17	
70-74	49	125	12	12	
75-79	45	81	11	8	
80+	45	76	11	7	
Sex (ref = male)	195	493	48	47	.660
Female	210	559	52	53	
Race (ref = non-Hispanic white)	396	1036	98	98	.356
Ethnic minority	9	16	2	2	
Preoperative alcohol use (ref = no)	160	296	41	29	<.001
Yes	232	738	59	71	
Preoperative tobacco use (ref = never)	166	524	41	50	.005
Quit	197	447	49	43	
Yes	40	72	10	7	
Charlson score (ref = 0)	219	697	54	66	<.001
1	77	179	19	17	
2+	108	176	27	17	
Year (ref = April-December 2011)	20	125	5	12	<.001
2012	44	189	11	18	
2013	97	207	24	20	
2014	112	180	28	17	
2015	117	192	29	18	
January-July 2016	15	159	4	15	
BMI preoperative mean (SD, range)	30.1 (6.8, 14.6 to 58.2)	29.1 (6.1, 15.6 to 56.4)			.010
BMI preoperative group (ref = normal, <25)	77	270	21	27	.094
Overweight, 25-29.99	131	357	35	36	
Obese, 30-34.99	88	198	24	20	
Severe obese, 35-39.99	49	107	13	11	
Morbid obese, 40+	25	59	7	6	
Length of stay (LOS), days mean (SD, range)	2.6 (1.6, 0 to 12)	1.9 (1.2, 0 to 13)			<.001
LOS, group (ref = <4 d)	321	982	79	93	<.001
>3 d	84	70	21	7	
Discharge disposition (ref = home)	303	922	75	88	<.001
Facility	102	130	25	12	
Surgery length, minutes (SD, range)	87 (32, 42 to 397)	86 (27, 47 to 270)			.812
Surgery length group (ref = 42-70 min)	139	274	34	26	<.001
71-90 min	139	469	34	45	
91-110 min	65	183	16	17	
111-400	61	126	15	12	
PCS preoperative mean (SD, range)	38.5 (6.4, 23.5 to 57.7)	40.7 (6.7, 23.5 to 67.7)			<.001
PCS preoperative group (ref = 50+)	25	114	7	11	<.001
40-49.99	96	356	25	35	
30-39.99	221	504	58	49	
20-29.99	37	54	10	5	
MCS preoperative mean (SD, range), n = 2209 (95%)	47.4 (8.9, 17.9 to 70.2)	50.4 (8.9, 17.9 to 70.2)			<.001
MCS preoperative group (ref = 60+)	30	137	8	13	<.001
50-59.99	122	420	32	41	
40-49.99	153	344	40	34	
<40	73	116	19	11	
PCS postoperative mean (SD, range)	46.7 (8.8, 26.7 to 67.7)	48.9 (8.7, 23.5 to 67.7)			<.001
PCS postoperative group (ref = 50+)	133	440	38	48	.002
40-49.99	117	304	33	33	
30-39.99	93	166	27	18	
<30	8	14	2	2	
PCS change (SD, range)	8.2 (7.8, −12.6 to 38.1)	8.1 (7.8, −15.3 to 32.3)			.798
PCS clinically significant improvement, >5 score increase (ref = no)	119	325	35	36	.963
Yes	217	589	65	64	
Latest PCS postoperative time period (ref = 0-45 d postoperative), n = 1417 (87%)	55	212	16	23	<.001
46-299 d postoperative	80	260	23	28	
300-420 d postoperative (1 y)	165	301	47	33	
421+ d postoperative	51	151	15	16	
Second primary THA? (ref = no)	374	964	92	92	.657
Yes	31	88	8	8	
Any-cause hip reoperation within 90 d postoperatively (ref = no)	402	1038	99	99	.347
Yes	3	14	1	1	

Percentages made not add up to 100 due to missingness or rounding.

BMI, body mass index; SD, standard deviation.

Older age, female sex, higher Charlson Score, lower PCS, lower MCS, and alcohol use were all associated with increased LOS (Table 2). Multivariate linear regression, adjusted for preoperative variables, demonstrated that the DAA was associated with shorter LOS by 0.49 days (95% confidence interval [CI] = 0.32-0.65, *P* < .001) (Table 2). From our initial sample, 154 patients (11%) had an extended LOS (>3 days) and were included in our analysis for this variable. Older age, female sex, higher Charlson Score, lower MCS, morbid obesity (BMI > 40), and alcohol use were all associated with an increased rate of extended LOS. After adjustment for confounding variables, multivariate logistic regression demonstrated the PA to be associated with extended LOS (odds ratio = 0.33, 95% CI = 0.21-0.50, *P* < .001) (Table 3).

**Table 2:** Multivariate linear regression model for whether the direct anterior approach is associated with a difference in length of stay (d) compared with the posterior approach for total hip arthroplasty.

Variable	LOS difference (d)	95% CI low	95% CI high	*P* value
Approach (ref = posterior)				
Anterior	−0.49	−0.65	−0.32	<.001
Age group (ref = <55)				
55-59	0.13	−0.06	0.33	.177
60-64	0.21	−0.01	0.43	.061
65-69	0.14	−0.06	0.33	.177
70-74	0.58	0.36	0.80	<.001
75-79	0.67	0.42	0.92	<.001
80 +	1.07	0.78	1.36	<.001
Sex (ref = male)				
Female	0.32	0.19	0.46	<.001
Charlson score (ref = 0)				
1	0.22	0.07	0.38	.005
2+	0.53	0.31	0.75	<.001
PCS (ref = 50+)				
40-49.99	0.03	−0.14	0.20	.726
30-39.99	0.23	0.03	0.43	.021
20-29.99	0.54	0.15	0.92	.006
MCS (ref = 60+)				
50-59.99	0.18	0.04	0.33	.015
40-49.99	0.32	0.16	0.49	<.001
<40	0.60	0.29	0.90	<.001
Alcohol use (ref = no)				
Yes	−0.17	−0.32	−0.02	.022

**Table 3:** Multivariate logistic regression model for whether the direct anterior approach is associated with a longer length of stay (at least 4 days) compared with the posterior approach for total hip arthroplasty.

Variable	Longer LOS OR	95% CI low	95% CI high	*P* value
Approach (ref = posterior)				
Anterior	0.33	0.21	0.50	<.001
Age Group (ref = <55)				
55-59	2.78	1.24	6.19	.013
60-64	1.99	0.90	4.40	.090
65-69	2.39	1.09	5.27	.030
70-74	4.33	1.99	9.42	<.001
75-79	3.79	1.58	9.07	.003
80+	7.23	3.04	17.15	<.001
Sex (ref = male)				
Female	1.67	1.08	2.59	.021
Charlson score (ref = 0)				
1	1.58	0.93	2.69	.093
2+	2.72	1.71	4.31	<.001
PCS (ref = 50+)				
40-49.99	0.51	0.17	1.48	.213
30-39.99	1.08	0.40	2.95	.877
20-29.99	1.89	0.59	6.06	.283
MCS (ref = 60+)				
50-59.99	5.22	1.22	22.36	.026
40-49.99	7.23	1.69	30.98	.008
<40	11.32	2.50	51.25	.002
BMI (ref = normal, <25)				
Overweight, 25-29.99	0.88	0.51	1.52	.658
Obese, 30-34.99	0.90	0.49	1.63	.727
Severe obese, 35-39.99	0.82	0.39	1.72	.597
Morbid obese, 40+	2.35	1.09	5.06	.029
Alcohol use (ref = no)				
Yes	0.60	0.39	0.91	.016

BMI, body mass index; OR, odds ratio.

Of the patients included, 232 (16%) were discharged to a facility. Older age, female sex, higher Charlson Score, lower PCS, lower MCS, morbid obesity, and alcohol use were all associated with facility discharge (Table 4). Multivariate logistic regression demonstrated that the DAA was associated with decreased odds of facility discharge (odds ratio = 0.54, 95% CI = 0.37-0.79, *P* < .001) (Table 4).

**Table 4:** Multivariate logistic regression model for whether the direct anterior approach is associated with facility discharge compared with the posterior approach for total hip arthroplasty.

Variable	Facility discharge OR	95% CI low	95% CI high	*P* value
Approach (ref = posterior)				
Anterior	0.54	0.37	0.79	.001
Age group (ref = <55)				
55-59	2.76	1.23	6.20	.014
60-64	2.49	1.13	5.46	.024
65-69	4.21	1.95	9.09	<.001
70-74	7.56	3.51	16.27	<.001
75-79	13.31	6.06	29.27	<.001
80+	38.08	16.86	86.02	<.001
Sex (ref = male)				
Female	1.86	1.27	2.73	.002
Charlson score (ref = 0)				
1	1.21	0.77	1.90	.397
2+	1.54	1.03	2.30	.034
PCS (ref = 50+)				
40-49.99	1.58	0.72	3.48	.257
30-39.99	1.64	0.74	3.65	.225
20-29.99	4.25	1.55	11.68	.005
MCS (ref = 60+)				
50-59.99	1.37	0.69	2.71	.368
40-49.99	2.12	1.05	4.28	.036
<40	3.39	1.46	7.86	.004
BMI (ref = normal, <25)				
Overweight, 25-29.99	0.67	0.42	1.06	.089
Obese, 30-34.99	0.76	0.45	1.29	.314
Severe obese, 35-39.99	0.94	0.49	1.83	.865
Morbid obese, 40+	2.57	1.24	5.36	.012
Alcohol use (ref = no)				
Yes	0.55	0.38	0.79	.001

BMI, body mass index; OR, odds ratio.

With regard to functional outcomes, of the 1457 surgeries, the preoperative response rates were 97% of patients in our sample having completed at least one of these outcome metrics. In the postoperative time periods, we had 87% of patients completing at least one of the metrics. Among patients with completed patient reported outcome measures both preoperatively and postoperatively, we had a capture rate of 86% for at least one of the metrics at both time points.

**Figure 1:**
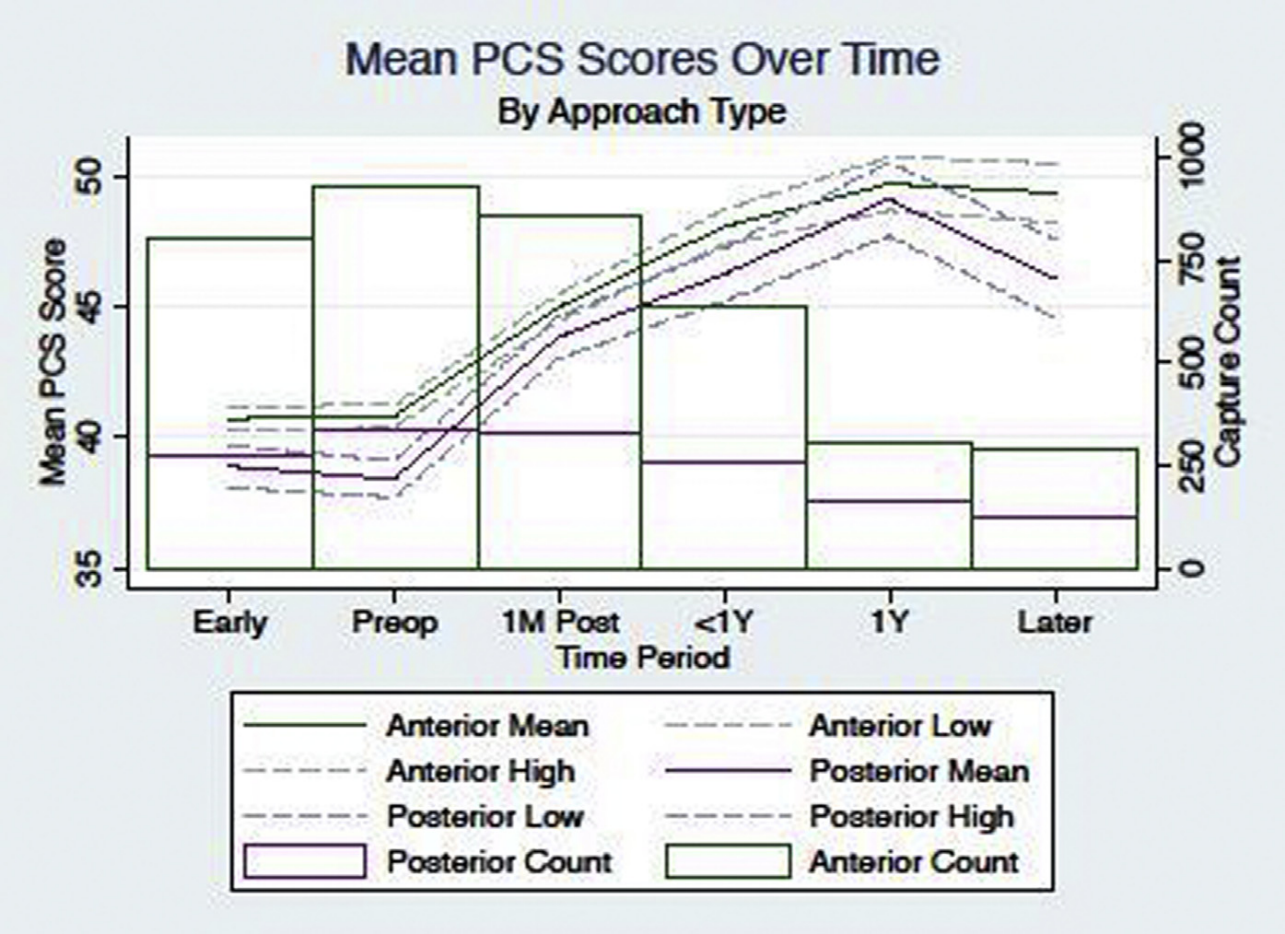
Change in physical component score over time for patients undergoing total hip arthroplasty through the direct anterior approach and posterior approach.

We found that DAA patients had a higher preoperative PCS score (40.7 vs 38.5, *P* < .001) and postoperative scores (48.9 vs 46.7, *P* < .001) than PA patients. Also, patients had higher preoperative MCS scores in the DAA cohort (DAA = 50.4, PA = 47.4, *P* < .001). However, there was no difference in the total change in PCS after THA between approaches (+8.1 vs +8.2; *P* = .798) (Fig. 1). Furthermore, there was no difference in percentage of patients who experienced clinically significant PCS change (*P* = .963).

In the multivariate linear regression model evaluating PCS improvement after THA, there was no significant difference between the DAA and PA (*P* = .542; Table 5). Similarly, there was no difference between approaches for odds of a clinically significant physical function improvement (*P* = .458) (Table 5). Those with a lower preoperative PCS score saw a significant improvement in their PCS score compared with those with a preoperative PCS score greater than 50, regardless of approach.

**Table 5:** Multivariate linear regression model for whether the direct anterior approach is associated with greater physical component score (PCS) improvement and odds of a clinically significant physical function improvement compared with the posterior approach for total hip arthroplasty.

Variable	PCS change	95% CI low	95% CI high	*P* value	Clinically sig. PCS OR	95% CI low	95% CI high	*P* value
Approach (ref = posterior)								
Anterior	0.30	−0.67	1.28	.542	1.13	0.82	1.54	.458

OR, odds ratio.

## Discussion

This study reports the findings of a multisurgeon comparison of prospective global HRQoL patient-reported outcomes for THA in patients undergoing the DAA vs the PA. The data demonstrate that both DAA and PA techniques for primary THA result in significant improvements in patient-reported physical function, as indicated by the PROMIS-10. The DAA patients presented with higher preoperative PCS and MCS scores as well as lower Charlson Comorbidity scores, suggesting a potential that patients with these characteristics may seek out surgeons who perform the DAA. However, there was no difference in the total change in PCS after THA between approaches or the percentage of patients who experienced a clinically significant PCS change. This demonstrates that regardless of approach, patients benefited similarly from THA at final follow-up.

Despite similar global improvement in function, our analysis demonstrated that the DAA was associated with shorter LOS and decreased odds of extended length of hospitalization >3 days after THA. Furthermore, the DAA was associated with decreased odds of discharge to a facility. Both these findings have been demonstrated in prior studies and are important considerations as LOS and post-hospital discharge disposition are important drivers of cost in THA [13,21,31]. In addition, despite some evidence suggesting increased reoperation rates with DAA THA, we did not identify any difference in 90-day reoperation rates between the 2 groups [32].

Multiple studies have reported on the short-term benefit of DAA THA in regard to faster recovery with fewer functional limitations, less dependence on assistive devices, and lower dislocation risk [[9], [10], [11], [12], [13], [14], [15], [16], [17], [18], [19], [20], [21]]. However, little data have been published on global HRQoL improvement after this approach. Rather, much of the current published literature reports outcomes pertaining to joint-specific PROs such as the Hip Disability and Osteoarthritis Outcome Score and Harris Hip Score. The first systematic review and meta-analysis comparing the DAA to the PA for THA published in 2015 also highlighted the lack of methodologically rigorous, prospective, trials with predefined reporting, standardized follow-up intervals, and outcome measures [20]. Only 2 included studies evaluated global HRQoL assessments, the SF-12 and SF-36, and owing to the heterogeneity of results, the authors were unable to provide a firm recommendation as to whether the anterior or PA was superior, as no study found a difference when comparing these global health metrics [6,17,33].

Other literature has reported varied results, with a prospective randomized control trial of 54 patients demonstrating the mini-posterior approach was superior in terms of the SF-12 mental scores at 3-week follow-up to the DAA. Conversely, the clinical significance of their findings is unclear as there were no differences found at later time points [34]. Improved early pain scores without difference in outcome scores between DAA and PA THA patients were reported in a single-surgeon randomized controlled trial by Christensen and Jacobs [20]. Patients were noted to have earlier discard of walking aides in the DAA group, yet neither of the SF-12 subscales demonstrated significant differences between groups after surgery [20]. In a single-surgeon retrospective review evaluating patient-reported physical function between DAA (86 patients) and PA (135 patients) THA patients, the VR-12 Physical and Mental Component Summary scores were assessed at 1 month, 3 months, and 1 year after surgery [6]. In that study, the DAA was associated with greater PCS improvement at 3 months than the PA, but there were no differences in adjusted PCS at either 1 month or 12 months. Finally, in a small retrospective review of 24 matched DA patients to 24 PA patients, at 3-month follow-up, the DAA group demonstrated significantly higher scores for the VR-12 Mental, VR-12 Physical, and SF-12 Physical scores [35]. There were no reported outcomes at any other time point reported, and how patients were selected for the DAA or PA was not clear, concerning for the possibility of selection bias. Our cohort is significantly larger than that of these prior studies looking at global HRQoL metrics, the present study is the only study using the PROMIS-10, and we believe the inclusion of multiple surgeons further enhances the applicability of our results.

It is important to note that the DAA patients, in addition to higher PCS and MCS scores at presentation, tended to be healthier and have slightly lower BMIs than the PA patients which also suggests a potential selection bias on patients seeking out DAA THA. As surgeons who routinely use only one approach for THA were included in the study and patients at our facility have the ability to choose their surgeon, this supports that some level of self-selection among patients toward the DAA might exist. There is currently no literature that we are aware of addressing this subject specifically, and further analysis of what factors may have played a role in this (sociodemographic variables, education level, health literacy, and so on) was beyond the scope of this study.

There are several limitations to the present study. The findings presented in our study were identified after adjustment for confounding clinical and demographic variables. However, despite the fact that all data were collected prospectively, the retrospective nature of our analysis precludes the ability to control for all confounding factors. In addition, some of the patients experiencing extended LOS may have been due to reasons other than medical, such as awaiting rehabilitation or skilled nursing facility availability. However, all surgeons in the study provided care from the same academic medical practice, limiting selection based on factors such as location and insurance status. Furthermore, while we controlled for the year of surgery, and we presume these trends would have affected patients from both surgical approach groups equally, this could confound our results. There were also more DAA hips in our cohort which is indicative of surgeon preference at our institution and could influence the results despite us controlling for confounders. We did exclude any surgeon who performed both approaches to try and avoid surgeon-patient selection bias. We also understand there may be bias toward rapid recovery of DAA patients, yet once this approach was adopted at our institution, all patients regardless of approach were treated by the same protocol.

## Conclusions

Despite the stated limitations, our analysis is the largest sample of HRQoL data from patients undergoing DAA THA by multiple surgeons. These data from our prospectively collected institutional registry provide further support to the association of the DAA with decreased LOS, extended LOS, and odds of facility discharge after surgery, compared with the PA. Our study also demonstrates that there was no difference in overall physical function improvement between the 2 surgical approach groups, with both DAA and PA patients experiencing significant improvement after THA. What is perhaps most interesting was the trend toward patients with higher HRQoL measures at baseline undergoing DAA THA which may represent a unique, previously unreported factor driving the popularity of this approach.

## Conflicts of interest

The authors declare the following financial interests/personal relationships which may be considered as potential competing interests: W. E. Moschetti is in the speakers' bureau or gave paid presentations for DePuy, Medscape, and Heraeus; is a paid consultant for DePuy; received research support from DePuy; received other financial or material support from Medacta; and is a board/committee member in New England Orthopaedic Society. B. J. Keeney is in the editorial board of Journal of Arthroplasty and the advisory board of Spine. D. Jevsevar has stock or stock options in Risalto Healthcare and is a board/committee member in AAHKS EBPC, AAOS DBT Committee, AAOS Bylaws Committee, and AAOS Registry Oversight Committee.

## Appendix A Supplementary data

Conflict of Interest Statement for Jevsevar

Conflict of Interest Statement for Keeney

Conflict of Interest Statement for Kunkel

Conflict of Interest Statement for Moschetti
