# Supplementary material for: Do Patients With Higher Preoperative Functional Outcome Scores Preferentially Seek Direct Anterior Approach Total Hip Arthroplasty?
Source: Arthroplast Today. 2021 Jun 22;10:6–11. doi: 10.1016/j.artd.2021.05.018 (PMC8226394; doi:10.1016/j.artd.2021.05.018)
Supplement: Conflict of Interest Statement for Jevsevar [file mmc1.pdf]

INDIVIDUAL CONFLICT OF INTEREST STATEMENT

American Association of Hip and Knee Surgeons

(Adopted from the American Academy of Orthopaedic Surgeons disclosure statement)

The following form **must be filled out completely and submitted by each author (example, 6 authors, 6 forms).**
All items require a response. If there is no relevant disclosure for a given item, enter "*None.*"

Manuscript Title: Do Patients with Higher Pre-operative Functional Outcome Scores Preferentially Seek Direct Anterior Approach Total Hip Arthroplasty?

1. Royalties from a company or supplier (The following conflicts were disclosed)
none

2. Speakers bureau/paid presentations for a company or supplier (The following conflicts were disclosed)
none

3A. Paid employee for a company or supplier (The following conflicts were disclosed)

none

3B. Paid consultant for a company or supplier (The following conflicts were disclosed)
none

3C. Unpaid consultants for a company or supplier (The following conflicts were disclosed)

none

4. Stock or stock options in a company or supplier (The following conflicts were disclosed)

Risalto Healthcare

5. Research support from a company or supplier as a Principal Investigator (The following conflicts were disclosed)
none

6. Other financial or material support from a company or supplier (The following conflicts were disclosed)
none

7. Royalties, financial or material support from publishers (The following conflicts were disclosed)

none

8. Medical/Orthopaedic publications editorial/governing board (The following conflicts were disclosed)

none

9. Board member/committee appointments for a society (The following conflicts were disclosed)
AAHKS EBPC
AAOS DBT Committee
AAOS Bylaws Committee
AAOS Registry Oversight Committee

Each author must sign AND print or type his/her name, date and submit a separate form
In addition, one BLINDED Conflict of Interest form (no author names used) should be submitted per manuscript with all author disclosures.

Author Name (Print or Type)	Author Signature	Date
David S. Jevsevar, MD, MBA	David S. Jevsevar	11/12/20
