# Supplementary material for: Do Patients With Higher Preoperative Functional Outcome Scores Preferentially Seek Direct Anterior Approach Total Hip Arthroplasty?
Source: Arthroplast Today. 2021 Jun 22;10:6–11. doi: 10.1016/j.artd.2021.05.018 (PMC8226394; doi:10.1016/j.artd.2021.05.018)
Supplement: Conflict of Interest Statement for Moschetti [file mmc4.docx]

# INDIVIDUAL CONFLICT OF INTEREST STATEMENT

***American Association of Hip and Knee Surgeons***

(Adopted from the American Academy of Orthopaedic Surgeons disclosure statement)

The following form **must be filled out completely and submitted by each author (example, 6 authors, 6 forms).**

**All items require a response. If there is no relevant disclosure for a given item, enter "*None*.”**

**Manuscript Title:** Do Patients with Higher Pre-operative Functional Outcome Scores Preferentially Seek Direct Anterior Approach Total Hip Arthroplasty?

1. Royalties from a company or supplier (The following conflicts were disclosed)

None

2. Speakers bureau/paid presentations for a company or supplier (The following conflicts were disclosed)

Depuy, Medscape, Heraeus

3A. Paid employee for a company or supplier (The following conflicts were disclosed)

None

3B. Paid consultant for a company or supplier (The following conflicts were disclosed)

Depuy

3C. Unpaid consultants for a company or supplier (The following conflicts were disclosed)

None

4. Stock or stock options in a company or supplier (The following conflicts were disclosed)

None

5. Research support from a company or supplier as a Principal Investigator (The following conflicts were disclosed)

Depuy

6. Other financial or material support from a company or supplier (The following conflicts were disclosed)

Medacta

7. Royalties, financial or material support from publishers (The following conflicts were disclosed)

None

8. Medical/Orthopaedic publications editorial/governing board (The following conflicts were disclosed)

None

9. Board member/committee appointments for a society (The following conflicts were disclosed)

New England Orthopaedic Society

**Each author must sign AND print or type his/her name, date and submit a separate form**

In addition, one BLINDED Conflict of Interest form (no author names used) should be submitted per manuscript with all author disclosures.

Wayne Moschetti
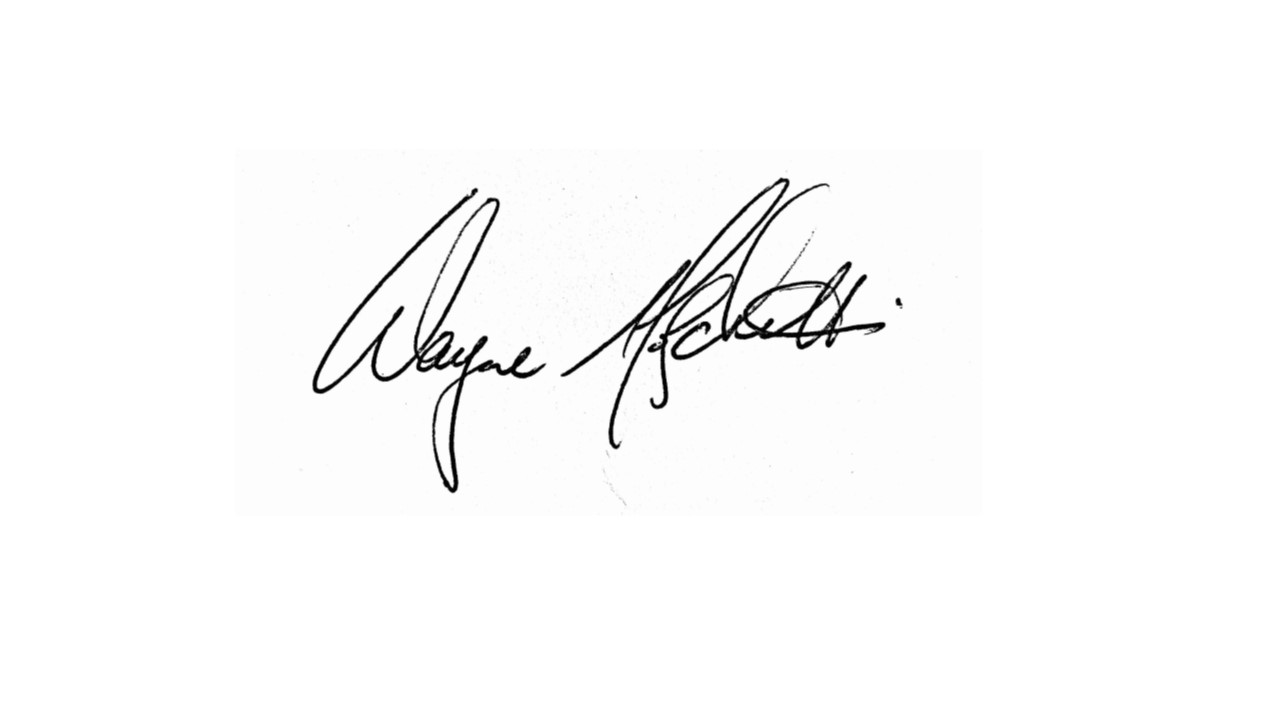
 10/31/2020

Author Name (Print or Type) Author Signature Date
